# Obstructive Jaundice Secondary to Choledocholithiasis With Chronic Cholecystitis

**DOI:** 10.7759/cureus.91041

**Published:** 2025-08-26

**Authors:** Sagarika B, Akshay V Gokak, Anil Bellad

**Affiliations:** 1 General Surgery, Jawaharlal Nehru Medical College, Belagavi, Belagavi, IND

**Keywords:** biliary obstruction, cbd stone, choledocholithiasis, geriatric surgery, mrcp, obstructive jaundice, open cholecystectomy

## Abstract

Obstructive jaundice, usually from choledocholithiasis, often coexists with chronic cholecystitis and poses higher risks in the elderly. Timely diagnosis with ultrasonography (USG)/magnetic resonance cholangiopancreatography (MRCP) and prompt intervention are critical. This case report highlights the diagnostic approach, surgical management, and outcomes in elderly patients with large common bile duct (CBD) stones. A 78-year-old man presented with jaundice, abdominal pain, and fever. MRCP revealed a large common bile duct (CBD) stone measuring 3.0 cm with features of cholecystitis, confirming obstructive jaundice secondary to choledocholithiasis. Additional workup, including hepatitis A virus/hepatitis E virus (HAV/HEV) serology, ruled out viral causes. The patient underwent open cholecystectomy with CBD exploration with stone extraction and T-tube placement. Intraoperatively, a sludge-filled gallbladder and dilated cystic duct were noted. Postoperative recovery was uneventful with the complete resolution of obstructive symptoms. Successful stone extraction with surgical management remains effective for large CBD stones in elderly patients. Timely surgical intervention resolved biliary obstruction, emphasizing the efficacy of open CBD exploration for complex choledocholithiasis in geriatric patients.

## Introduction

Obstructive jaundice secondary to choledocholithiasis is a frequent presentation in surgical practice, yet the presence of giant common bile duct (CBD) stones (>3 cm) is rare, particularly in elderly patients. The coexistence of chronic calculous cholecystitis, dilated cystic duct, and large impacted CBD stones poses unique diagnostic and therapeutic challenges [1]. Timely imaging with magnetic resonance cholangiopancreatography (MRCP) or ultrasonography (USG) and surgical intervention are crucial to prevent serious complications such as cholangitis and pancreatitis [2]. While endoscopic retrograde cholangiopancreatography (ERCP) and laparoscopic CBD exploration are now widely accepted first-line interventions, their success rates diminish significantly in cases of large, impacted, or irregular stones, especially when accompanied by gallbladder pathology and altered ductal anatomy [3,4].

In elderly patients, comorbidities, tissue fragility, and limited physiological reserve further complicate management, making the choice of intervention critical [5,6]. Minimally invasive approaches, including lithotripsy (mechanical, pneumatic, or laser), can fragment large stones; however, procedural time, multiple sittings, and incomplete clearance remain concerns, especially in stones exceeding 2.0-2.5 cm [7,8].

We report a case of a 78-year-old man with a giant CBD stone (3.0 × 2.0 × 1.8 cm), contracted gallbladder, dilated cystic duct (19 mm), and chronic calculous cholecystitis, managed successfully via single-stage open cholecystectomy with CBD exploration and T-tube drainage. This report highlights the importance of individualized surgical decision-making, the role of MRCP in preoperative planning, and the continued relevance of open exploration in select complex cases where minimally invasive options are less feasible.

## Case presentation

A 78-year-old man was admitted to Karnatak Lingayat Education Society's (KLES) Dr. Prabhakar Kore Hospital and Medical Research Centre, Belagavi, on 14 January 2025 for the evaluation and management of obstructive jaundice. He presented with a one-month history of progressive jaundice and two months of epigastric pain radiating to the right hypochondrium aggravated by food intake. Associated symptoms included the yellowish discoloration of the skin and sclera, two episodes of fever in the preceding three days, one episode of non-bilious vomiting, dark urine, and pale stools. There was no history of alcohol use, blood transfusions, or prior biliary disease.

On examination, the patient was conscious, oriented, and icteric. Abdominal examination revealed a pendulous abdomen with tenderness in the right hypochondrium without palpable organomegaly. Other systemic examinations were unremarkable.

Laboratory investigations showed elevated total bilirubin (11.4 mg/dL), direct bilirubin (2.65 mg/dL), and alkaline phosphatase (160 IU/L), suggestive of obstructive pathology. Hepatitis A virus (HAV IgM) and hepatitis E virus (HEV IgM) serology were negative.

**Figure 1:**
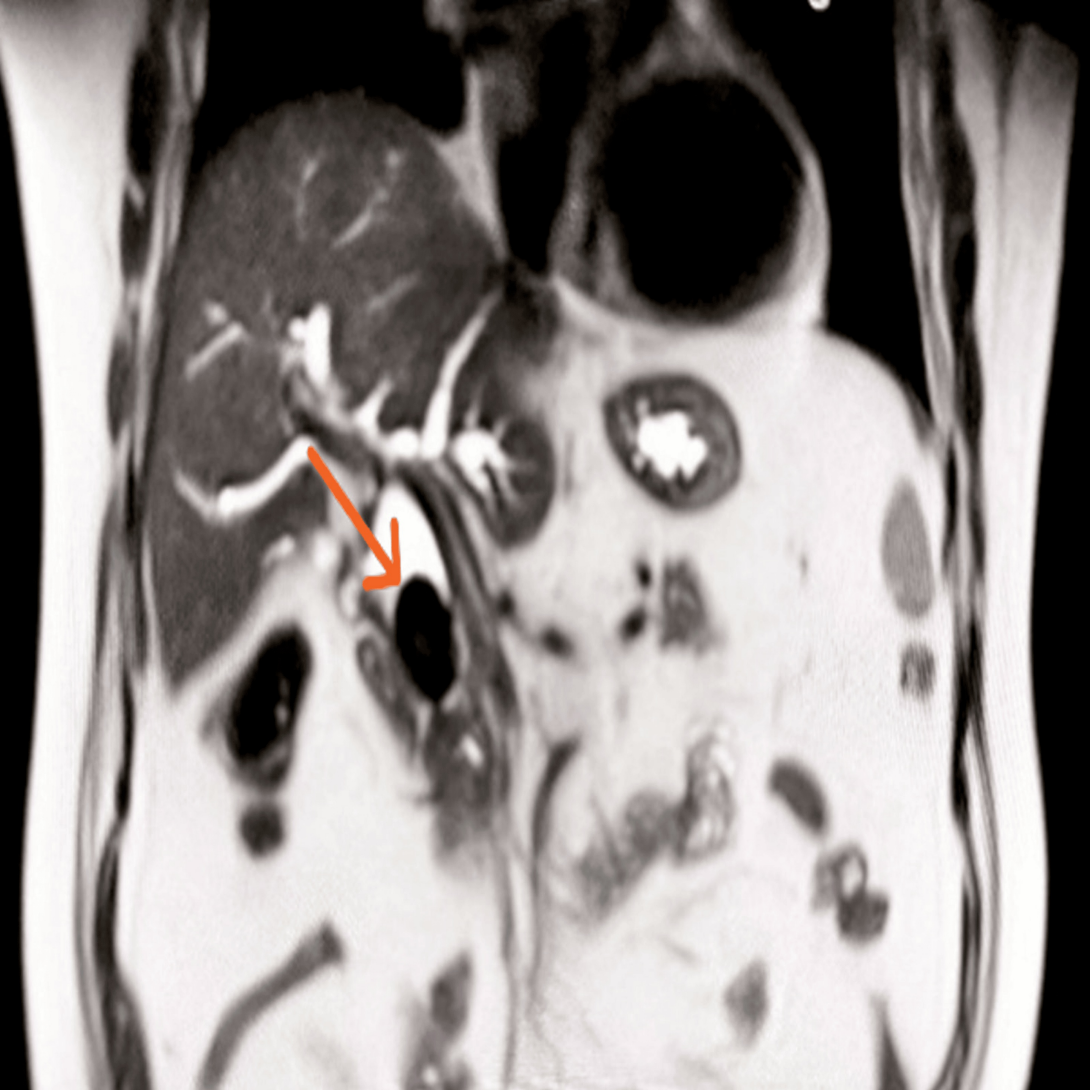
Coronal T2-weighted MRI image showing an intraductal filling defect in the region of the mid common bile duct (CBD), suggestive of an intraductal calculus

**Figure 2:**
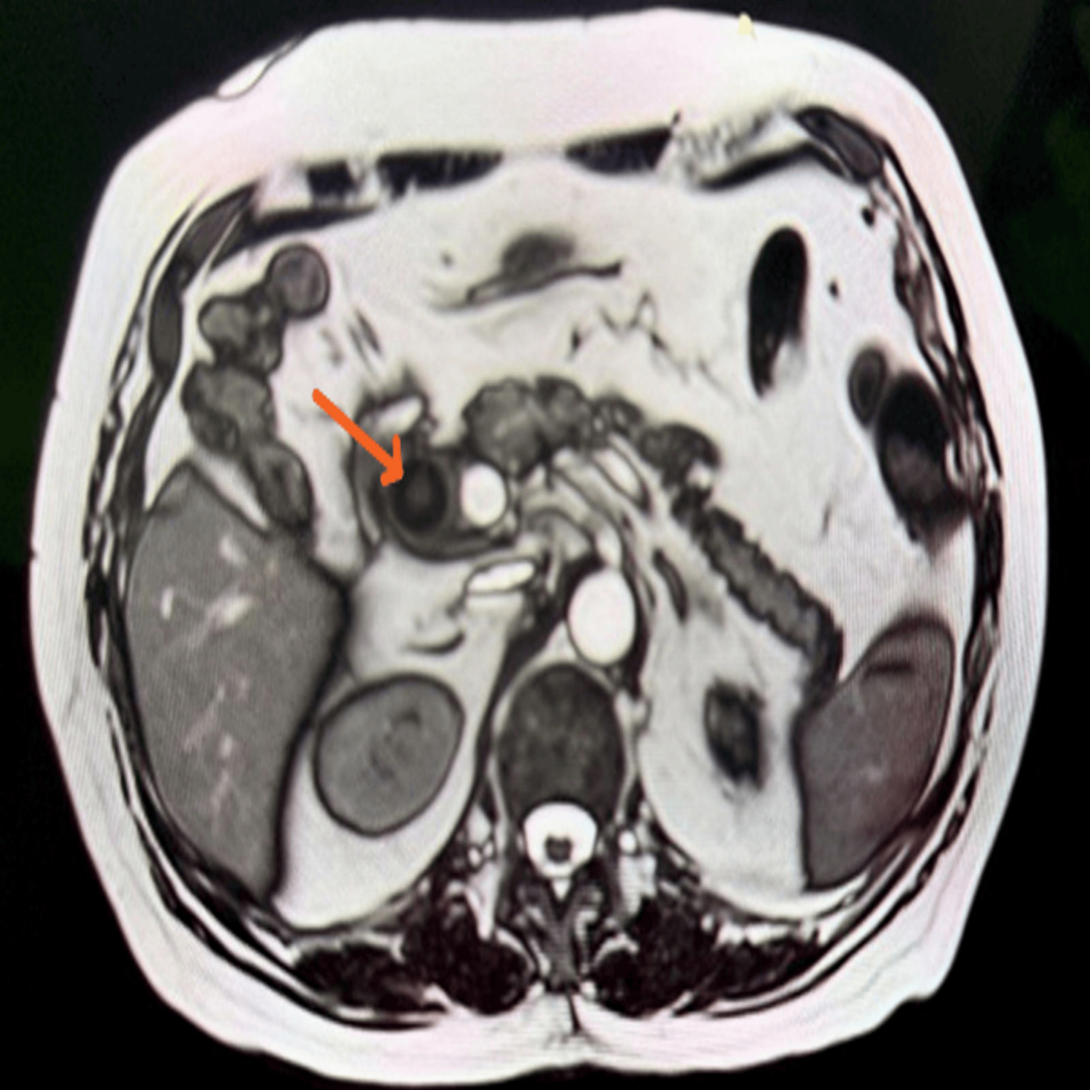
Axial T2-weighted MRI image showing an intraductal filling defect in the region of the mid common bile duct (CBD), suggestive of an intraductal calculus

**Figure 3:**
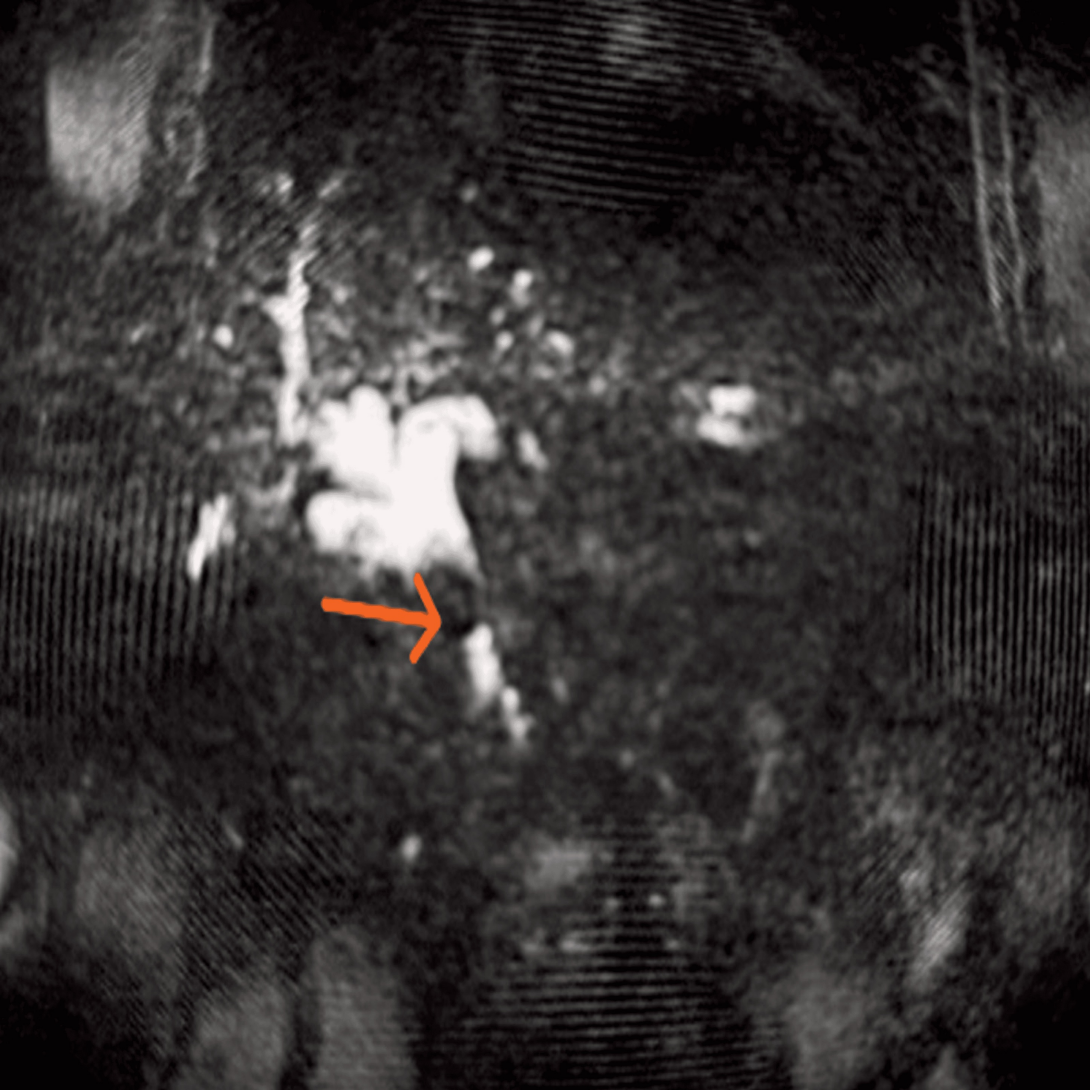
Magnetic resonance cholangiopancreatography (MRCP) shows a bright biliary tree with a central filling defect seen in the mid common bile duct region, suggestive of a calculus

The ultrasound of the abdomen showed a dilated proximal common bile duct (CBD) measuring 7.0 mm, mild biliary radicle dilatation, and sludge/microcalculi in the gallbladder (Figures 1, 2). MRCP confirmed a large CBD stone measuring 3.0 × 2.0 × 1.8 cm with moderate dilatation of the common hepatic duct (18 mm), right and left hepatic ducts (10 mm and 15 mm, respectively), and a small contracted gallbladder with a 3 mm calculus at the neck (Figure 3). The cystic duct was dilated (19 mm) with sludge; the pancreas appeared normal. A diagnosis of obstructive jaundice secondary to choledocholithiasis with chronic calculous cholecystitis was made.

**Figure 4:**
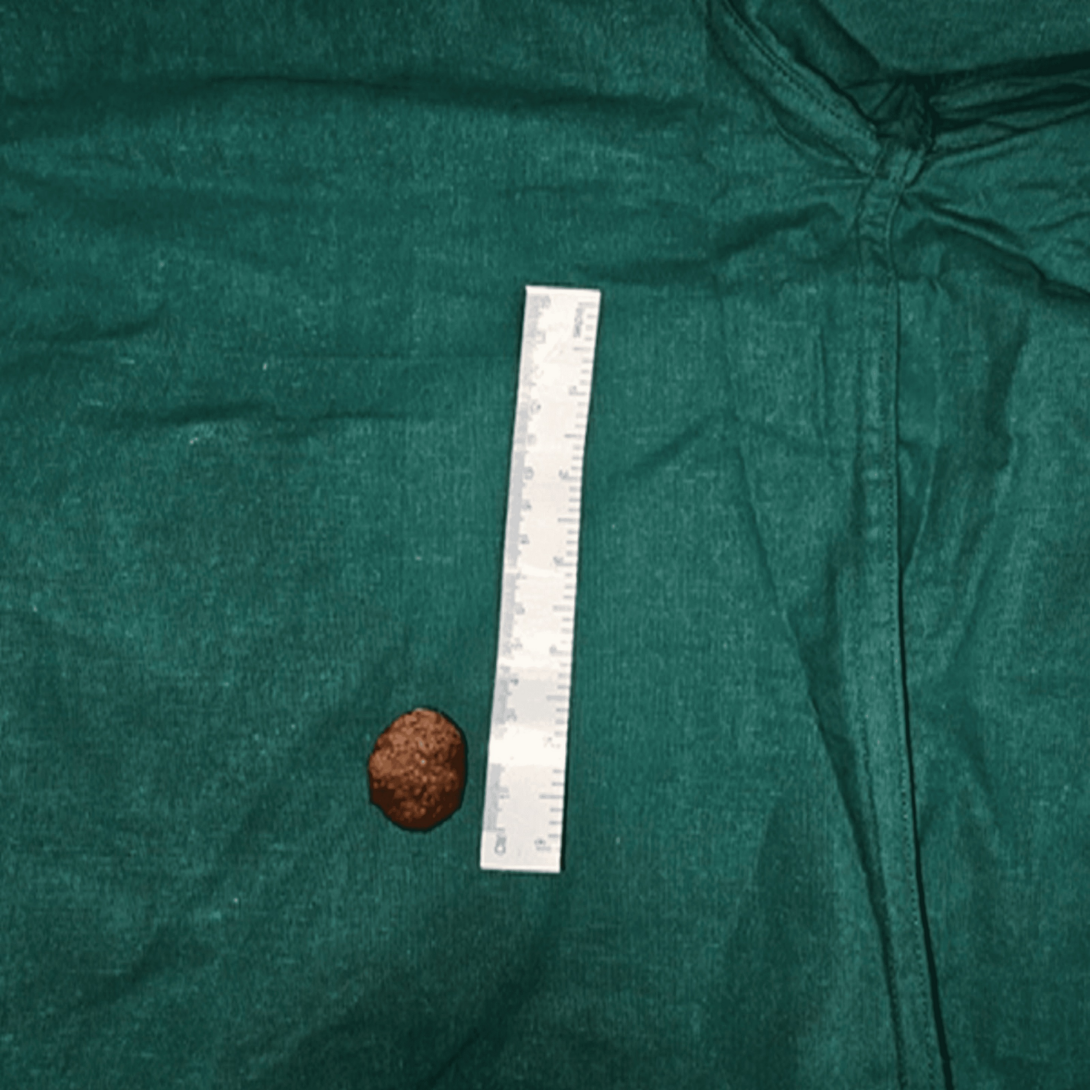
Gross specimen: large gallstone post cholecystectomy

The patient underwent open cholecystectomy with CBD exploration under general anesthesia. Intraoperative findings included a thick-walled, sludge-filled gallbladder with an impacted stone in the neck region, resulting in a dilated cystic duct and a large CBD stone measuring 3.0 × 2.0 cm with moderate dilatation of the common hepatic duct and right and left hepatic ducts. CBD stone was successfully extracted, and an 18 Fr T-tube was placed for biliary drainage (Figure 4). The procedure was well-tolerated with minimal blood loss. Postoperative recovery was uneventful, with the resolution of jaundice and abdominal symptoms confirming the effectiveness of the surgical management.

## Discussion

Obstructive jaundice in elderly patients presents a multifaceted diagnostic and therapeutic challenge, particularly when secondary to choledocholithiasis [4]. In this case, a 78-year-old man exhibited classical symptoms of biliary obstruction, including progressive jaundice, right upper quadrant pain, dark urine, pale stools, and constitutional symptoms such as fever and vomiting. The absence of a prior biliary history or hepatotoxic exposure prompted a focused evaluation toward mechanical causes. Imaging played a pivotal role in diagnosis. Initial ultrasonography suggested proximal CBD dilatation with gallbladder sludge, raising suspicion for distal obstruction. Magnetic resonance cholangiopancreatography (MRCP), a noninvasive and highly sensitive imaging modality, confirmed a large CBD stone measuring 3.0 × 2.0 × 1.8 cm and the associated dilatation of the common hepatic and intrahepatic ducts. MRCP also revealed a contracted gallbladder with a 3 mm neck calculus and sludge-filled cystic duct consistent with chronic calculous cholecystitis. These findings ruled out hepatocellular or viral causes of jaundice, supported further by negative HAV and HEV serology, and confirmed a mechanical etiology.

Choledocholithiasis, especially with stones larger than 1.5-2.0 cm, is associated with increased failure rates for endoscopic extraction via ERCP, particularly when complicated by gallbladder disease, impacted stones, or altered anatomy [2]. In this patient, stone size (>3.0 cm), dilated cystic duct (19 mm), and associated gallbladder inflammation warranted definitive surgical management. Open cholecystectomy with CBD exploration, though more invasive than laparoscopic or endoscopic options, offered the best therapeutic approach under the given anatomical and clinical circumstances.

Intraoperative findings of a thick-walled, sludge-filled gallbladder with an impacted stone confirmed the chronicity of inflammation and obstruction. Surgical stone extraction, followed by T-tube drainage, allowed the effective decompression of the biliary tree and ensured postoperative monitoring through T-tube cholangiography if required. The patient's stable intraoperative and postoperative course with rapid resolution of jaundice and abdominal pain demonstrates the efficacy and safety of this approach even in elderly individuals.

This case exemplifies the importance of individualized management in elderly patients with complex biliary pathology. While laparoscopic cholecystectomy remains the standard for gallstone disease, technical difficulties posed by giant stones, inflamed tissue planes, and the risk of incomplete clearance in endoscopic procedures justify opting for open surgery in select cases. Literature also supports open CBD exploration as a viable and often necessary option in large stone burden or failed ERCP scenarios, particularly in high-risk patients where repeated interventions may increase morbidity [4].

Furthermore, the early recognition of obstructive features and prompt imaging, specifically MRCP, allowed for precise surgical planning and minimized the risk of complications such as ascending cholangitis, biliary sepsis, or pancreatitis [8]. Given the increasing prevalence of biliary tract disease in ageing populations, this case underscores the importance of a timely structured approach combining clinical suspicion, imaging, and appropriate surgical intervention tailored to the patient's profile.

Limitations

This is a single-patient report, so the findings lack generalizability. No comparator (ERCP/laparoscopic options) was included, limiting treatment comparison. Case reports are low-level evidence and cannot establish causation [9].

## Conclusions

This case highlights the successful surgical management of obstructive jaundice due to a giant CBD stone in an elderly patient through open cholecystectomy with CBD exploration and T-tube drainage. Preoperative MRCP was pivotal in delineating anatomy, confirming the diagnosis, and guiding operative planning.

While minimally invasive methods remain the standard for most biliary stones, open CBD exploration should be considered in select cases, particularly with giant, impacted stones and distorted anatomy or when complete clearance is uncertain. This case emphasizes the importance of individualized treatment strategies and demonstrates that open surgical management, when appropriately indicated, can achieve excellent outcomes with minimal morbidity even in geriatric patients.
